# Efficacy of enteral nutrition in patients with Crohn’s disease on maintenance anti-TNF-alpha antibody therapy: a meta-analysis

**DOI:** 10.1007/s00535-019-01634-1

**Published:** 2019-10-22

**Authors:** Fumihito Hirai, Teruyuki Takeda, Yasumichi Takada, Masahiro Kishi, Tsuyoshi Beppu, Noritaka Takatsu, Masaki Miyaoka, Takashi Hisabe, Kenshi Yao, Tosiharu Ueki

**Affiliations:** 1 Inflammatory Bowel Disease Center, Fukuoka University Chikushi Hospital, Chikushino, Fukuoka Japan; 2 Department of Gastroenterology, Fukuoka University Faculty of Medicine, 7-45-1, Nanakuma, Jonan-ku, Fukuoka, 814-0180 Japan; 3 Department of Gastroenterology, Fukuoka University Chikushi Hospital, Chikushino, Fukuoka Japan

**Keywords:** Crohn’s disease, Enteral nutrition, Anti-TNF-alpha antibody, Meta-analysis

## Abstract

Enteral nutrition (EN) is effective in Crohn’s disease (CD) patients and has been shown to have an inhibitory effect on loss of response to anti-tumor necrosis factor (TNF)-alpha antibody therapy; however, the current level of evidence is not sufficient. The objective of this meta-analysis was to determine whether EN in combination anti-TNF-alpha antibody therapy is useful in maintaining remission. PubMed was used to identify all relevant studies. A total of nine articles were identified including one randomized control trial, two prospective cohort studies, and six retrospective cohort studies. We performed a meta-analysis on all these articles to assess the remission maintenance effect of EN (*n* = 857). The remission or response maintenance effect in the EN group was 203/288 (70.5%), which was higher than 306/569 (53.8%) in the non-EN group. The odds ratio for long-term remission or response using fixed effects model and random effects model were 2.23 (95% CI 1.60–3.10) and 2.19 (95% CI 1.49–3.22), respectively. The usefulness of EN was unclear in two prospective studies that were conducted immediately after remission induction with anti-TNF-alpha antibody therapy was detected. Differences in the definition of relapse and the observation period among articles were considered to be limitations. This analysis suggests that EN is effective for maintaining remission in patients already in remission or response as a result of anti-TNF-alpha antibody maintenance therapy.

**Electronic supplementary material:**

The online version of this article (10.1007/s00535-019-01634-1) contains supplementary material, which is available to authorized users.

## Introduction

Crohn’s disease (CD) is a chronic inflammatory bowel disease and tends to follow a progressive course with the development of bowel complications such as stricture and fistula over time [1–3]. The cause of CD remains unknown, but the involvement of dietary antigens has been known, and dietary habits such as excessive fat intake are suggested in both onset and relapse [4–7]. Therefore, implementing enteral nutrition (EN) consisting mainly of amino acids and peptides and limiting the total amount of oral intake and fat intake by patients with CD can, as a consequence, reduce dietary antigens and improve the pathology of CD [8, 9]. EN has also other mechanisms linked to the preventive effects such as decreasing mucosal cytokines [10], correction of gut permeability [11], and modification of gut flora [12]. Furthermore, obesity and visceral fat are regarded as risk factors for the onset and exacerbation of CD [13, 14], and well-balanced nutrition augmented by EN can assist in their reduction. Therefore, dietary restriction or EN is widely recognized as effective treatments in patients with CD, and also patients noticed it [15]. Exclusive enteral nutrition (EEN) with elemental, semi-elemental diets and polymeric formulations are used in pediatric patients as first-line therapy for remission induction and its use is recommended in many guidelines [16–18]. In contrast, the use of EN is usually limited in adult CD patients, including patients with complicated short bowel syndrome or intestinal dysfunction after extensive digestive tract resection [19]. However, in Asia, especially in Japan, it is considered that EEN is an effective treatment for patients with active CD as a remission induction therapy [20, 21]. Moreover, EN is established as a remission maintenance therapy and several papers reported the efficacy of EN in various situations for patients with CD [22–24]. Currently, the use of anti-tumor necrosis factor (TNF)-alpha antibodies has become mainstream because of their high efficacy in CD [25–29]. According to the recent nationwide cohort study, parallel to an increasing use of thiopurines and anti-TNF-alpha antibody in inflammatory bowel disease (IBD) over time, a persistent significant decrease in surgery rates was confirmed [30]. On the other hand, despite the use of anti-TNF-alpha antibody of IBD, surgery is still required in 30–40% of patients with CD during the maintenance therapy [31]. Hence, considering the long-term outcome of CD, loss of response (LOR) due to the lower blood trough level and the emergence of anti-drug antibody (ADA) are problematic [32–34]. These have been addressed by combination therapy with immunomodulators (IMMs) or dose increases, or switching to different classes of drugs [35, 36], but long-term safety and medical economic issues have been pointed out [37, 38]. In fact, the patients tend to accept elevated severe adverse effect (AE) risks in exchange for clinical efficacy; however, they are not able to accept even mild AE risks if the treatment efficacy is lower or uncertain [39]. In recent years, reports indicating that EN can enhance the therapeutic effect and suppress LOR of anti-TNF-alpha antibody agents have been attracting attention [40–45]. Implementation of EN itself is inherently extremely safe, although there are minor concerns such as osmotic diarrhea. Since EN is not a drug, it is not necessary to consider the possible exacerbation of side effects due to interactions with concomitantly administered drugs. In addition, since the mechanism of action is different from the other treatments mentioned above, an add-on effect can be expected [46]. This meta-analysis was performed to determine whether EN in combination with anti-TNF-alpha antibody therapy is useful.

## Materials and methods

### Literature search

A literature search on PubMed was conducted for articles published by October 31, 2018, using the following search formula: (Crohn’s disease OR Crohn disease) AND (elemental diet OR enteral nutrition OR polymeric diet) AND (infliximab OR adalimumab OR certolizumab pegol OR golimumab OR TNF-alpha inhibitor).

### Study selection and exclusion

Two authors (F.H and T.T) reviewed the results of the literature search independently. Inclusion criteria were defined as follows: (1) anti-TNF-alpha antibody is used as maintenance therapy (at least 16 weeks) in adult CD patients, (2) clinical remission or response maintenance effect is compared between patients who received EN and patients who did not receive EN (the dose of EN was not taken into consideration) and (3) the number of event occurrences is clearly described both for the EN and non-EN groups. Exclusion criteria were defined as follows: studies of diseases other than CD (e.g., ulcerative colitis and inflammatory bowel disease unclassified), abstracts without full texts, case reports, reviews and pediatric studies.

### Statistical analysis

Point estimates of the odds ratio for long-term remission (EN group/non-EN group) and their 95% confidence intervals were determined for each article and overall for all articles. Two models were applied as statistical models for the common odds ratio of all articles with respect to long-term remission: a fixed effects model, which is a model where literature effects are not considered as variables, and a random effects model, which is a model where literature effects are considered as variables. The methods used to estimate the common odds ratio were the Mantel–Haenszel method for the fixed effects model and the DerSimonian–Laird method for the random effects model [47, 48]. The Breslow–Day test was performed as a test of heterogeneity between articles in assessing the odds ratio for long-term remission [49]. The null hypothesis is “The true value of the odds ratio for each article is the same among all articles”. Statistically significant heterogeneity between articles was defined as *P* < 0.05. In addition, the Higgins and Thompson's *I*^2^ statistic was calculated as a measure of heterogeneity [50]. The statistical software SAS ver.9.4 (SAS Institute, Cary, NC, USA) was used for analysis. Statistical analyses were performed by an independent third party, AC Medical Inc. (Tokyo, Japan).

## Results

### Study selection

**Fig. 1:**
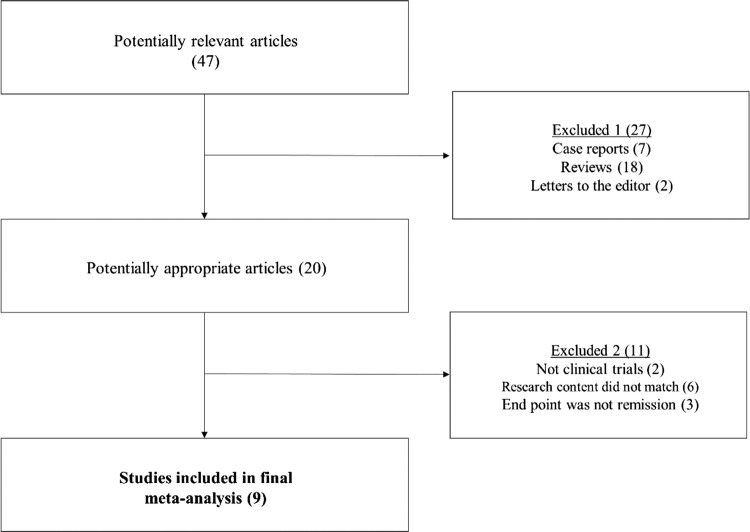
Algorithm demonstrating article search of this meta-analysis. Finally, nine studies were included in this study

The initial literature search identified a total of 47 articles. From the articles, seven case reports, 18 reviews, and two letters to the editor were excluded. Of the remaining 20 articles, two more were excluded as they were not clinical trials, six because the research content did not match (infliximab vs enteral nutrition monotherapy: 1, infliximab without enteral nutrition: 4, infliximab + enteral nutrition vs conventional therapy: 1), and three articles where the end point was not clinical remission or response. Based on the results of these exclusions, nine articles meeting all criteria were included in this meta-analysis [40–45, 51–53] (Fig. 1). In two of the articles [52, 53], the number of relapse events was not accurately described, but shown only in a graph. We, therefore, confirmed the number of events by communicating with the author via email.

### Details of selected studies

The selected articles included one randomized control trial (RCT), two prospective observational cohort studies and six retrospective observational cohort studies. After all the total number of this meta-analysis contained 857 patients. The type of enteral formulation used was elemental diet (ELENTAL^®^, EA Pharma Co., Ltd, Tokyo, Japan) only in seven articles and semi-elemental diet and polymeric formulations also included in two articles. The type of anti-TNF-alpha antibody was infliximab (IFX) alone in six articles, one of which included patients treated at 10 mg/kg. In addition, adalimumab (ADA) alone was used in one article, and both IFX and ADA were used in two articles. The definition of EN group and relapse or LOR, and the review period varied depending on the article (Table 1). Only 3 out of 9 studies mentioned dose of EN intake in detail (Table 1). All selected studies were from Japan. The dose escalation of IFX (10 mg/kg every 8 week) and the shortening of administration of IFX (5 mg/kg every 4–7 week) were approved in 2011 and 2017, respectively, in Japan. The dose escalation of ADA (80 mg every other week) was approved in 2016. The shortening of administration of ADA is not approved yet. Therefore, indication and treatment strategy on dose escalation and shortening of anti-TNF-α antibody were different among the selected articles depending on the time periods conducted the study (shown in Supplementary Table 1).

**Table 1:** Summary of studies including EN in meta-analysis

Ref. no.	Author	Study design	Group	Therapy for Crohn's disease	Average dose of EN	Total number	Long-term remission or response		Definition of clinical remission or response	(%) Duration of follow-up period
Year		Randomization					Number	Percent		
[40]	Tanaka et al.	Retrospective	EN +	IFX (5 mg/kg) + EN (> 3762 kJ/day)	NA	51	30	58.8	Reduction in HBI score, or number of fistula	16 weeks after IFX last dose
2006		Observational	EN −	IFX (5 mg/kg) without EN	NA	59	22	37.3	Increase in HBI score, or number of fistula	16 weeks from IFX last dose
[41]	Yamamoto et al.	Prospective	EN +	IFX (5 mg/kg) + EN (≥1200 kcal/day)	NA	32	25	78.1	CDAI score < 150	56 weeks after remission
2010		Observational	EN −	IFX (5 mg/kg) without EN	NA	24	16	66.7	CDAI score ≥ 150	56 weeks from remission
[42]	Sazuka et al.	Retrospective	EN +	IFX (5 mg/kg) + EN (≥ 600 kcal/day)	NA	29	23	79.3	CDAI score < 150	Last visit in follow-up
2012		Observational	EN −	IFX (5 mg/kg) + EN (<600 kcal/day)	NA	45	22	48.9	CDAI score ≥ 150, etc	Median follow-up from remission: 85 weeks
[43]	Hirai et al.	Retrospective	EN +	IFX (5 mg/kg) + EN (≥ 900 kcal/day)	1233 ± 62 (mean ± SE)	45	31	68.9	CRP < 0.3 mg/dL	Last visit in follow-up
2013		Observational	EN −	IFX (5 mg/kg) + EN (< 900 kcal/day)	535 ± 32 (mean ± SE)	57	24	42.1	CRP ≥ 1.5 mg/dL, etc	Mean follow-up from remission EN + group: 525.3 days (75.0 weeks) EN− group: 558.9 days (79.8 weeks)
[44]	Kamata et al.	Retrospective	EN +	IFX (5 mg/kg) + EN (≥ 900 kcal/day)	1246 ± 350 (mean ± SE)	28	27	96.4	Clinical remission	Last visit in follow-up
2015		Observational	EN −	IFX (5 mg/kg) + EN (< 900 kcal/day)	142 ± 238 (mean ± SE)	97	77	79.4	Clinical symptoms, etc	Mean follow-up from remission EN + group: 799 days (114 weeks) EN- group: 771 days (110 weeks)
[45]	Hirai et al.	Prospective	EN +	IFX (5 mg/kg)/ADA (40 mg) + EN (≥ 900 kcal/day prescribEN)	NA	37	24	64.9	CDAI score < 200	Last visit in follow-up
2018		Observational	EN −	IFX (5 mg/kg)/ADA (40 mg) only	NA	35	22	62.9	CDAI score ≥ 200	2 years (104 weeks) from IFX/ADA first dose
[51]	Hisamatsu et al.	Prospective	EN +	IFX (10 mg/kg) + EN (900–1200 kcal/day)	NA	14	11	78.6	CDAI score < 150	Last visit in follow-up
2018		Randomized	EN −	IFX (10 mg/kg) without EN	NA	6	3	50.0	Termination of maintenance therapy	56 weeks form IFX 10 mg/kg dose
[52]	Moroi et al.	Retrospective	EN +	IFX (5 mg/kg)/ADA (40 mg) + EN (≥ 900 kcal/day)	NA	27	13	48.1	Clinical remission	Last visit in follow-up
2018		Observational	EN −	IFX (5 mg/kg)/ADA (40 ng) + EN (< 900 kcal/day)	NA	154	75	48.7	Clinical relapse	At most 10 years (521 weeks) from IFX first dose
[53]	Sugita et al.	Retrospective	EN +	ADA (40 mg) + EN (≥ 900 kcal/day)	1044 ± 192 (mean ± SD)	25	19	76.0	HBI score < 5	Last visit in follow-up
2018		Observational	EN −	ADA(40 mg) + EN (< 900 kcal/day)	88 ± 200 (mean ± SD)	92	45	48.9	HBI score ≥ 5, etc	Mean follow-up from remission EN + group: 1282 days (183 weeks) EN − group: 1340 days (191 weeks)

*IFX* infliximab, *NA* not applicable, *HBI* Harvey–Bradshaw Index, *ADA* adalimumab, *CDAI* Crohn's Disease Activity Index, *EN* elemental nutrition, *CRP* C-reactive protein

### Enteral nutrition during anti-TNF-alpha inhibitor and LOR risk

**Fig. 2:**
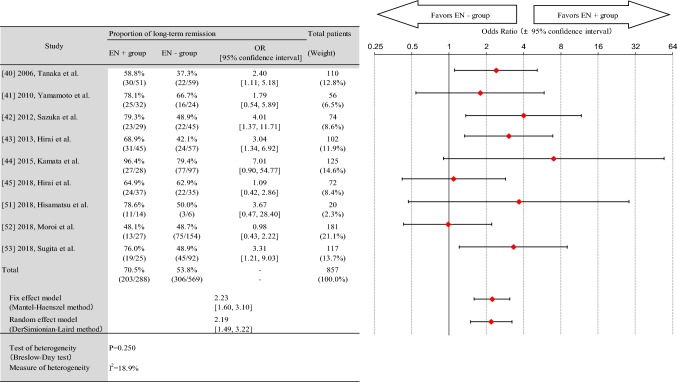
Forest plot of odds ratio for long-term remission. The odds ratio for long-term remission using fixed effects model and random effects model was 223 (95% CI 160–310) and 219 (95% CI 149–322), respectively

The remission maintenance effect in the EN group was 203/288 (70.5%), which was higher than 306/569 (53.8%) in the non-EN group. Figure 2 presents a forest plot of the odds ratios (OR) for long-term remission. The pooled OR of EN for clinical remission or response maintenance was 2.23 [95% confidence interval (CI) 1.60–3.10] in the fixed effects model and 2.19 [95% CI 1.49–3.22] in the random effects model. The results of the heterogeneity test showed no statistically significant heterogeneity (*P* = 0.250). The measure of heterogeneity was at a relatively low level (*I*^2^ = 18.9%).

### Publication bias

**Fig. 3:**
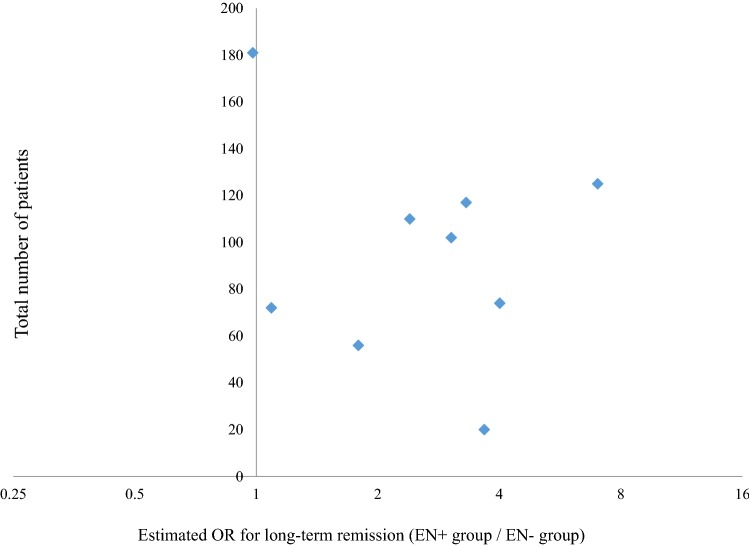
Funnel plot of odds ratio for long-term remission

A high publication bias was suggested by a funnel plot representing the article-by-article point estimates of the odds ratio for long-term remission on the horizontal axis and the number of patients (total for both the EN group and non-EN group) for each article on the vertical axis (Fig. 3).

## Discussion

Many cases of Crohn’s disease (CD) eventually require surgery and relapse after surgery is common. CD is regarded as a disease that causes progressive disability [2]. However, recently, it has been reported that this disease course can be changed by introducing effective medical treatment with an anti-TNF-alpha antibody at an appropriate time [30, 54, 55]. While anti-TNF-alpha antibody therapy not only improves symptoms but also induces mucosal healing in many cases, LOR often occurs during the maintenance therapy [32, 33]. If LOR occurs, measures that take blood concentration and anti-drug antibodies into account are recommended [35, 36]. In addition, in IFX, which is highly immunogenic, combination with IMMs is known to improve the therapeutic effect and reduce the risk of LOR [56, 57]. EN does not directly induce an increase in anti-TNF-alpha antibody blood concentration or reduce antibody production. However, an increasing number of recent reports have shown how the use of EN in combination with an anti-TNF-alpha antibody is clinically useful due to the add-on effect of EN itself [40–45, 51–53]. In the present review, in which these studies were meta-analyzed, the pooled OR for maintenance of remission or relapse with EN was 2.23 in the fixed effects model and 2.19 in the random effects model. In other words, the meta-analysis suggested that combination with EN improves the remission or response maintenance effect of treatment with an anti-TNF-alpha antibody. However, it remains unclear on the dose of EN, adherence to EN, the timing of starting EN for getting more combination efficacy. Hisamatsu et al. concluded that combination with EN at over 1200 kcal was useful in patients with LOR among those patients where the IFX dose had already been doubled [51]. Except for this limited indication, it was not possible to identify the patients who would benefit from EN in combination with medical therapy or to clarify the required dose in this review. This was due to variations in the clinical backgrounds of the target patients and the dose of nutritional therapy despite the lowest dose being set at 600 kcal. Since it is difficult to continue EN over a prolonged period, it is practically impossible to conduct RCTs to examine the therapeutic effect of EN. One RCT was included and only three prospective studies were included in this meta-analysis. In retrospective studies, patients who were able to continue receiving EN for a prolonged period are analyzed as the EN group, enabling assessment of the effect of EN combination therapy; however, recruitment bias cannot be ruled out. According to the report by Hirai et al., although patient adherence was confirmed in the EN group prior to the enrollment in accordance with the protocol for the prospective cohort, only 29.7% were able to continue with EN at the targeted calorific content of 900 kcal over a 2-year period [45]. The authors considered that the low adherence in the EN group was the main reason why the efficacy of combination therapy with EN was not demonstrated. Measures to improve adherence, such as alleviating amino acid odor, are needed. In the two prospective trials, the subjects were patients who achieved clinical remission by anti-TNF-alpha antibodies in early treatment phase and were subsequently divided into the EN group and the non-EN group. These trials did not include patients who were in clinical remission by means of maintenance phase of anti-TNF-alpha antibodies. Therefore, the administration period of TNF-alpha antibodies was relatively short and the majority of the subjects were naive to biologics. These factors might be influenced on the result of failing to confirm the usefulness of concomitant EN. On the contrary, in the other trials examined mainly for the patients with maintenance therapy of anti-TNF-alpha antibodies as the subjects, it was suggested that concomitant EN with anti-TNF-alpha antibody was effective for preventing relapse.

In fact, there is another meta-analysis, which was published in Nguyen et al. in 2015, on current topic [58]. They reported that specialized EN therapy with IFX resulted in 109 of 157 (69.4%) patients reaching clinical remission compared with 84 of 185 (45.4%) with IFX monotherapy [OR 2.73; 95% CI 1.73–4.31, *p* < 0.01]. The slight difference between the results of these two meta-analyses was presumed to be related to the type of anti-TNF-α antibodies (subjects of meta-analysis by Nguyen et al. were administered only IFX), the number of included papers and the timing of publications.

This review has several limitations. First, as mentioned above, the backgrounds of target patients and the definitions of relapse are different. Many of the studies reviewed used a retrospective cohort as the study design and there are only a few high quality studies. Second, all the studies adopted for meta-analysis were conducted in Japan and it cannot be confirmed if the results of these studies can be extrapolated to other regions. Third, publication bias exists. The remission maintenance rate is generally high because the target in all articles was limited to patients who used anti-TNF-alpha antibody therapy. The articles are associated with the interventional use of EN in combination with medical therapy, and this could lead to the conclusion that EN has an add-on effect or at the very least is comparable. And last but least, the issue on the therapeutic drug monitoring and anti-drug antibody were not analyzed because all studies did not mention these topics.

In conclusion, EN in combination with anti-TNF-alpha antibody therapy can help to prevent the incidence of clinical relapse including LOR in maintenance therapy. The combined therapy may affect the better course, for example, to extend clinical remission or response. Although the required dose of EN is unknown, doses of at least 600–900 kcal have been cited in reports in which efficacy was demonstrated. There is a possibility that EN appears to be more strongly indicated in CD patients with non-colonic type whose dose of an IFX has already been doubled due to LOR. Prospective studies with a high level of evidence need to be conducted worldwide in the future.

## Electronic supplementary material

Below is the link to the electronic supplementary material.

Supplementary file1 (DOCX 29 kb)
