# Supplementary material for: Efficacy of enteral nutrition in patients with Crohn’s disease on maintenance anti-TNF-alpha antibody therapy: a meta-analysis
Source: J Gastroenterol. 2019 Oct 22;55(2):133–41. doi: 10.1007/s00535-019-01634-1 (PMC6981109; doi:10.1007/s00535-019-01634-1)
Supplement: Supplementary file 1 [file 535_2019_1634_MOESM1_ESM.docx]

Supplementary Table 1. Indication and treatment strategy on dose escalation and shortening of anti TNF-α antibody in selected studies

Ref. No, author, year	Dose escalation of anti TNF-α antibody at baseline	Shortening of anti TNF-α antibody at baseline	Definition of LOR or relapse	Anti TNF-α antib optimization strat at LOR or relaps
[40] Tanaka et al. 2006	NA	NA	clinical activity (HBI)<br>additional therpy	NA
[41] Yamamoto et al. 2009	NA	NA	clinical activity (CDAI)	NA
[42] Sazuka et al. 2012	NA	NA	clinical activity (CDAI)<br>CRP	dose escalation<br>shortening of II
[43] Hirai et al. 2013	NA	NA	CRP<br>additional therpy<br>shortening of IFX	shortening of IFX (li
[44] Kamata et al. 2015	included	included	clinical symptom<br>dose escalation of IFX<br>shortening of IFX	dose escalation<br>shortening of II
[45] Hirai et al. 2018	included	included	clinical activity (CDAI)<br>dose escalation of IFX, ADA<br>shortening of IFX, ADA<br>additional therpy	dose escalation<br>shortening of IFX,
[51] Hisamatsu et al. 2018	NA	NA	clinical activity (CDAI)	dose escalation of
[52] Sugita et al. 2018	NA	NA	clinical activity (HBI)<br>additional therpy	NA
[53] Moroi et al. 2019[3)]	included	included	dose escalation of IFX, ADA<br>shortening of IFX, ADA<br>additional therpy	dose escalation<br>shortening of IFX,

NA: not applicable

LOR: loss of response
IFX: infliximab, ADA: adalimumab,
HBI: Harvey-Bradshaw index,
CDAI: Crohn's disease activity index
